# Dengue Infections Among Household Contacts of Symptomatic Index Cases: Implications for Community-Based Intervention Studies

**DOI:** 10.3390/v17060859

**Published:** 2025-06-17

**Authors:** Erik Koehne, Liesbeth Van Wesenbeeck, Martin L. Hibberd, Annemie Buelens, Michiko Toizumi, Kim De Clerck, Leen Vijgen, Ole Lagatie, Lucy Masdin, Hien Anh Thi Nguyen, Hoang Huy Le, Duc Anh Dang, Mai Kim Huynh, Lien Thuy Le, Trieu Bao Nguyên, Stephane Hue, Hung Thai Do, Guillermo Herrera-Taracena, Freya Rasschaert, Lay-Myint Yoshida

**Affiliations:** 1 Department of Pediatric Infectious Diseases, Institute of Tropical Medicine, Nagasaki University, Nagasaki 852-8523, Japan; erik.koehne@nagasaki-u.ac.jp (E.K.); toizumi@nagasaki-u.ac.jp (M.T.); 2 Johnson & Johnson, 2340 Beerse, Belgium; lvwesenb@its.jnj.com (L.V.W.); lvijgen@its.jnj.com (L.V.);; 3 Faculty of Infectious and Tropical Diseases, London School of Hygiene and Tropical Medicine, London WC1E 7HT, UKstephane.hue@lshtm.ac.uk (S.H.); 4 School of Tropical Medicine and Global Health, Nagasaki University, Nagasaki 852-8523, Japan; 5 National Institute of Hygiene and Epidemiology, Hanoi 100000, Vietnam; 6 Pasteur Institute in Nha Trang, Nha Trang 650000, Vietnam; mai064@yahoo.com (M.K.H.); lethuylien250786@gmail.com (L.T.L.);; 7 Johnson & Johnson, Horsham, PA 19044, USA; 8 Graduate School of Biomedical Sciences, Nagasaki University, Nagasaki 852-8523, Japan

**Keywords:** dengue, infection, household contacts, community, Vietnam

## Abstract

Background: Dengue is a global health concern, with half of the world’s population at risk and no antiviral treatment available. This Phase 0 study investigated dengue infections among household contacts (HHCs) of dengue index cases (ICs) and assessed the feasibility of conducting a Phase 2 trial for a novel antiviral. Methods: Participants were enrolled in Nha Trang, Vietnam, from April 2022 to February 2023. Dengue ICs were identified within 72 h of fever onset, and their healthy adult HHCs enrolled within 48 h. Blood samples and questionnaires were collected bi-weekly for four weeks, with a follow-up visit on day 40. DENV RT-qPCR, NS1, and anti-DENV IgM/IgG ELISAs were performed. Results: Overall, 130 dengue ICs and 301 HHCs were enrolled, with 91.7% (276/301) completing all follow-up visits. Baseline anti-DENV IgG showed prior dengue infections in 262/301 HHCs (87.0%). Fifty HHCs were excluded from the HHC infection analysis based on evidence of a DENV infection (viral load [VL], NS1, IgM, and IgG results) at enrollment. During follow-up, 2.0% of HHCs (5/251) had DENV infections based on virological parameters (DENV RNA and/or NS1 positivity), and anti-DENV IgG/IgM seroconversion was detected in 7.2% (18/251). Conclusions: This study demonstrated the operational feasibility of a dengue IC-HHC design for a Phase 2 trial.

## 1. Introduction

Dengue is a global public health threat, putting half of the world’s population at risk and causing 100–400 million cases annually [1]. Dengue is caused by the dengue virus (DENV), which consists of four distinct serotypes (DENV-1 to DENV-4) and is transmitted to humans during the feeding of an infected *Aedes* mosquito. Approximately 80% of dengue cases are asymptomatic, while in symptomatic cases, the first signs typically appear 2 to 7 days after infection, coinciding with the onset of viremia. In symptomatic cases, DENV RNA and the DENV NS1 antigen can be detected during the first 7 days of illness [2]. In the case of a primary dengue infection, anti-DENV IgM antibodies generally become detectable 4–5 days after symptom onset, while anti-DENV IgG antibodies appear after IgM, between days 7 and 14, and can be detected for many months and even years after infection. In post-primary DENV infections, a rapid increase in anti-DENV IgG titers is observed while the IgM response is low or even absent. Individuals with clinical symptoms typically experience an acute, self-limiting febrile illness that resolves within 4 to 7 days, including both dengue with warning signs (DWWS) and dengue without warning signs (DWoWS). However, approximately 1% of reported DENV infections result in severe dengue (SD) outcomes [3].

Asymptomatic and presymptomatic dengue infections have been shown to play a key role in dengue transmission dynamics, leading to outbreaks and epidemics [4,5,6]. Factors contributing to their significant role in dengue transmission include their higher prevalence relative to symptomatic DENV infections, the presence of DENV viremia, required for mosquito infection, also occurring in pre- and asymptomatic individuals, and the lack of apparent symptoms, allowing these individuals to maintain their daily activities and move between various locations. Investigation into dengue intervention strategies aimed at preventing both symptomatic and asymptomatic infections would be valuable for their potential to impact dengue disease burden as well as dengue transmission in the community.

To date, there is no dengue-specific antiviral treatment available, although a limited number of direct-acting antiviral strategies for treatment or prevention are currently in clinical development, including Johnson & Johnson’s mosnodenvir (Phase II), Novartis’ EYU688/NITD-688 (Phase II), and Visterra’s monoclonal antibody VIS513 (Phase II), currently licensed to the Serum Institute of India Pvt. Ltd. [7,8,9,10]. Two dengue vaccines—Dengvaxia^®^ (CYD-TDV) and Qdenga^®^ (TAK-003)—have been licensed, and a third, the Butantan–Dengue Vaccine, is in late-stage clinical development [11]. However, in 2024, Sanofi Pasteur announced that the manufacturing of Dengvaxia^®^ will be discontinued [12]. The use of Qdenga^®^ is recommended by the WHO in settings with high dengue transmission intensity. Until the efficacy–risk profile for DENV-3 and DENV-4 in seronegative people has been more thoroughly assessed, the WHO does not recommend the programmatic use of Qdenga^®^ in low to moderate dengue transmission settings [13].

To evaluate the potential of an anti-DENV antiviral agent for preventing dengue infections in the community, a study design that assesses feasibility and efficacy is essential. To this end, we conducted a prospective community-based study among household contacts (HHCs) of both hospitalized and non-hospitalized dengue index cases (ICs). We assessed the operational feasibility of a community-based IC-HHC study design, along with participant adherence to intensive sampling for endpoint evaluation. We evaluated the incidence of DENV infections using viral (DENV RNA or NS1) and serological markers (anti-DENV IgM/IgG) and examined disease characteristics along with the dynamics of viral and immunological responses in human hosts (ICs and HHCs).

## 2. Methods

### 2.1. Study Design

This Phase 0 study is a prospective community-based study on the HHCs of dengue NS1- and/or IgM-positive ICs. HHCs were enrolled and followed up twice a week for 4 weeks, with an additional follow-up on day 40 (Figure S1). The study was conducted in Nha Trang, Khanh Hoa Province, central Vietnam [14,15]. There are five government polyclinics, each covering 5 to 6 communes, providing outpatient treatment to a total of 27 communes. As a standard of care, all dengue cases that require hospitalization are referred to Khanh Hoa Tropical Diseases Hospital (TDH). The dengue NS1- and/or IgM-positive ICs were enrolled either at TDH or polyclinics covering all dengue patients visiting public health care settings at the study site. Participants were enrolled between April 2022 and February 2023.

### 2.2. Study Participant Enrollment

#### 2.2.1. Dengue Index Cases

Clinically suspected dengue cases aged 1–65 years who visited TDH and polyclinics within 72 h after the onset of illness (fever) were screened using the DENV NS1 and IgM/IgG rapid dual test (SD BIOLINE Dengue Duo rapid test) for positivity (NS1 and/or IgM) for inclusion in the study. Clinical and epidemiological information, along with a blood sample, was collected at enrollment. For hospitalized ICs, a second blood sample was collected at the time of hospital discharge.

#### 2.2.2. Household Contacts

The HHCs, aged 18–65 years, were contacted and enrolled within 48 h of IC enrollment. HHCs were defined as people living near the ICs, such as those spending the night under the same roof or sharing cooking facilities. The HHCs could not have any signs or symptoms of dengue infection at the time of enrollment. The enrolled HHCs were followed up and blood samples were taken twice a week for 4 weeks and on day 40, for a total of up to 10 visits (Figure S1).

### 2.3. Data and Sample Collection

The collected blood samples from dengue ICs and HHCs were transported to the Nagasaki University research laboratory at the Pasteur Institute in Nha Trang for sample processing and storage. A health diary was also provided to the HHCs to record their daily health status during the study period.

### 2.4. Sample Testing

Dengue antigen (NS1) and antibody testing (anti-DENV IgM/IgG) were performed using commercially available enzyme-linked immunosorbent assay (ELISA) test kits (EUROIMMUN Medizinische Labordiagnostika AG, Lübeck, Germany). Test results were interpreted following the manufacturer’s instructions. The DENV NS1 result was considered positive if the result was ≥11 relative units (RU)/mL and negative when <11 RU/mL. A positive anti-dengue IgM test result was defined as a ratio of the extinction of the sample to the calibrator ≥1.1; the result was borderline (indeterminate) if the ratio was ≥0.8 to <1.1 and negative if <0.8. Anti-dengue IgG was considered positive if the ELISA test result was ≥22 RU/mL and negative if <22 RU/mL. The detection of DENV RNA in serum was evaluated using a DENV real-time reverse transcription polymerase chain reaction (RT-PCR) from the LightMix^®^ Dengue Virus EC kit (Roche Diagnostics International AG, Risch-Rotkreuz, Switzerland); a cycle threshold < 35 was interpreted as a positive result; a cycle threshold ≥ 35 was considered negative. For a selected set of samples (the available viral load-positive samples), DENV serotype was assessed using a serotype-specific quantitative DENV RT-qPCR performed at Cerba Research (Cerba Research, Rijswijk, The Netherlands).

### 2.5. Case Definitions

ICs with baseline anti-DENV IgG levels < 22 RU/mL in the anti-DENV IgG ELISA were defined as primary DENV infections and ICs with baseline IgG levels ≥ 22 RU/mL as post-primary DENV infections. For HHCs, baseline anti-DENV IgG levels < 22 RU/mL classified HHCs as dengue-naïve at enrollment versus having a history of prior dengue exposure when their baseline IgG levels were ≥22 RU/mL.

The incidence of DENV infections in HHCs during the study period was evaluated based on viral and/or serological markers. Viral markers were evaluated from visit 2 onwards for the detection of post-baseline infections. As serology responses occur later in the infection, both visit 1 and visit 2 serology test results were considered as baseline, and serology markers to detect post-baseline infections were evaluated from visit 3 onwards. A DENV infection in a HHC was identified during the trial participation if the HHC met at least one of the following criteria: (1) a positive DENV RNA and/or NS1 test result from study visit 2 and onwards; (2) in the case of baseline negativity for anti-DENV IgG and/or IgM: a positive anti-DENV IgG and/or IgM test result from study visit 3 and onwards; (3) in the case of positive baseline anti-DENV IgG: a ≥2-fold increase in levels compared to baseline starting from study visit 3 and onwards. However, when a HHC had a positive DENV RNA and/or a positive NS1 test result at baseline (visit 1) and/or anti-DENV IgM was positive/indeterminate at baseline (visit 1 and/or visit 2) and/or had anti-DENV IgG seroconversion at visit 2, the HHC was considered to have a baseline DENV infection and was not included in the DENV infection follow-up analysis. Indeterminate anti-DENV IgM values at follow-up in HHCs were considered negative. Samples with high IgG levels at the first two visits and an increase of less than two-fold were diluted and retested to confirm the change in IgG levels.

### 2.6. Ethics

The study was approved by the ethics committee of Nagasaki University (No. 210916264) and the National Institute of Hygiene and Epidemiology (NIHE), Hanoi (No. 3985/QD-BYT). Written informed consent was obtained from the individuals to participate in the study. For dengue ICs aged 1–17 years, informed consent was obtained from a parent or guardian. The study execution and biological samples used in the project were reviewed and approved to ensure compliance with Consensus ethical principles derived from international guidelines (Declaration of Helsinki, Council for International Organizations of Medical Sciences (CIOMS) International Ethical Guidelines, applicable International Council for Harmonization (ICH) Good Clinical Practice (GCP) Guidelines, and applicable laws).

### 2.7. Data Availability

The study data will be shared upon request on a collaborative basis, with approval from the study investigators and sponsor. Personal information will be removed from the shared data.

## 3. Results

### 3.1. Study Disposition

**Figure 1:**
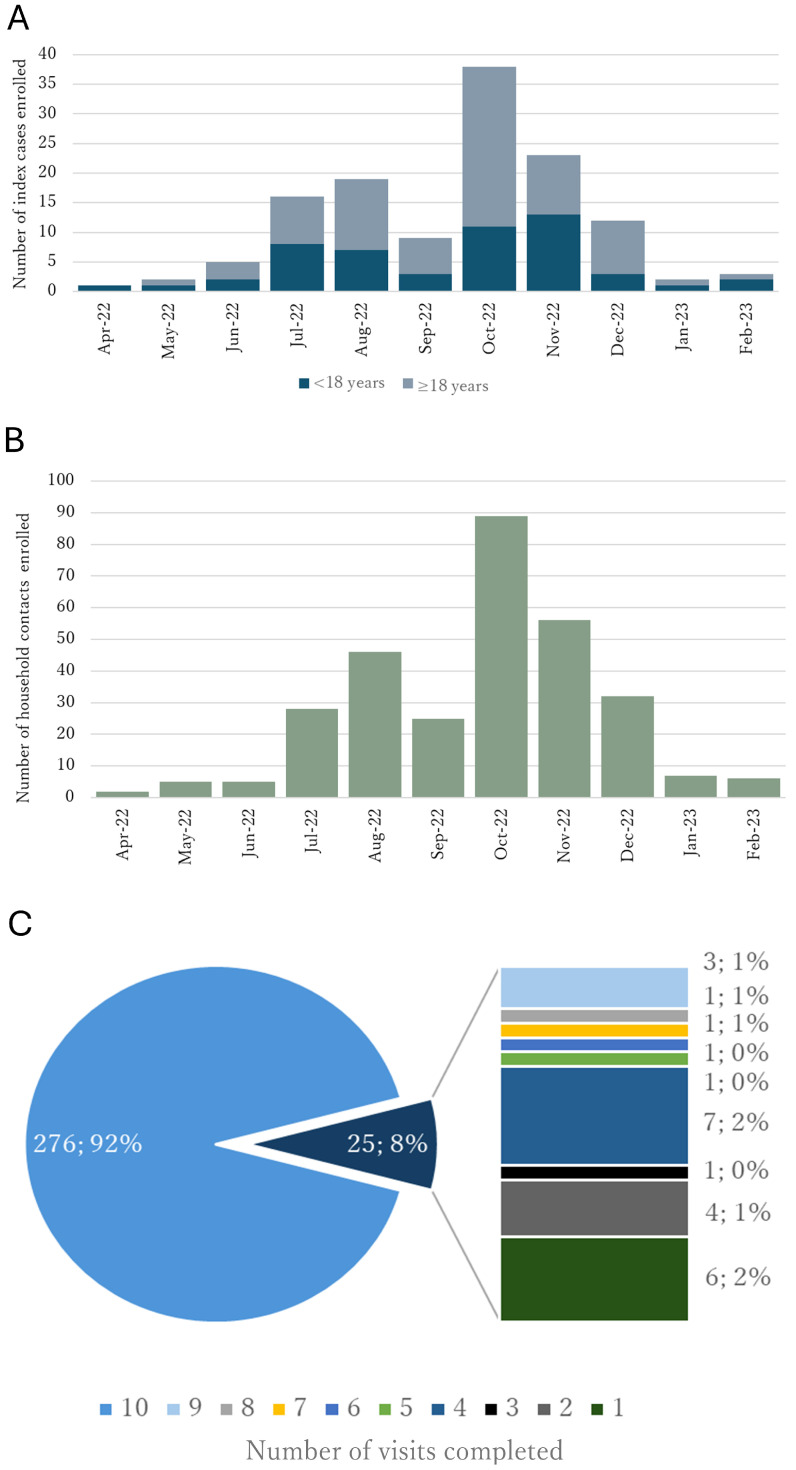
Enrollment of dengue index cases (**A**) and their household contacts (**B**), and the study completion rate for the household contacts (**C**). Study completion is defined as the successful collection of the scheduled blood samples and associated epidemiological data at each visit. The number of visits corresponds to the total completed by each study participant.

During the enrollment period between April 2022 and February 2023, a total of 130 ICs were enrolled in the study, with 40% (n = 52) of the ICs < 18 years of age (Figure 1A). The highest number of enrollments occurred during the period between July 2022 and December 2022. A total of 301 HHCs were enrolled (Figure 1B). The average number of HHCs per IC was 2.3.

### 3.2. Operational Feasibility of Index Case—Household Contact Study Design in Vietnam

The time between the onset of symptoms and the NS1/anti-DENV IgM/IgG rapid diagnostic test (RDT) was available for 129 ICs with a mean of 41.4 h. Except for 5 out of 129 ICs (3.9%), all ICs were screened using the RDT within 72 h after symptom onset, and for the majority of ICs, the RDT was performed within 48 h after symptom onset (92/129; 71.3%).

A high study completion rate was achieved with 92% of the HHCs (276/301) completing all 10 planned enrollment and follow-up visits (Figure 1C).

### 3.3. Characteristics and Dengue Infections in Index Cases

**Figure 2:**
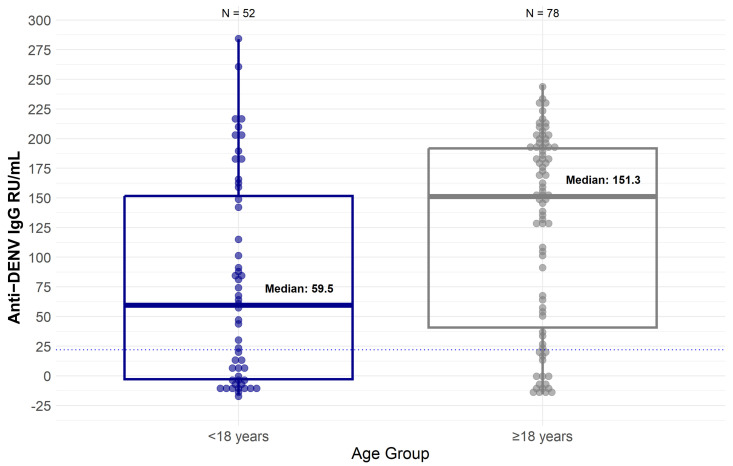
Box plot of anti-DENV IgG levels of index cases by age group at enrollment. The dotted line represents the cut-off for anti-DENV IgG seropositivity, set at 22 RU/mL.

The median age of the enrolled ICs was 23 years (IQR: 11–34), 10 years (IQR: 8–14) among the ICs < 18 years of age, and 31 years (IQR: 25–39) among ICs ≥ 18 years of age. Furthermore, 53.1% (69/130) of the ICs were male (Table 1). At enrollment, anti-DENV IgG titers were negative for 37 ICs (28.5%), indicating that those ICs had a primary DENV infection. Proportionally, more primary DENV infections were observed in the ICs < 18 years of age (21/52 (40.4%)) when compared to the ICs ≥ 18 years of age (16/78 (20.5%)) (Table 1). The remaining 93 ICs (71.5%) were anti-DENV IgG-positive at enrollment (post-primary DENV infections). At baseline, ICs < 18 years of age had a median anti-DENV IgG level of 59.5 RU/mL (IQR: −3.5 to 154.4), while those ≥18 years of age had a median of 151.3 RU/mL (IQR: 37.8 to 191.8) (Figure 2).

For most ICs (75.4%, 98/130), the rapid test was positive for NS1 only; 11.5% (15/130) of ICs were positive for both NS1 and IgM, and 13.1% (17/130) of ICs were only positive for IgM. The positive DENV NS1 and/or anti-DENV IgM/IgG rapid test result could be confirmed by DENV RT-qPCR (DENV RNA-positive Cp < 35) for 78 ICs (60%) and by NS1 ELISA for 81 ICs (62.3%). Serotype data at enrollment were obtained for 64 DENV RNA-positive ICs. The majority of DENV infections were caused by DENV-2 (51.6% [33/64]), followed by DENV-1 (40.6% [26/64]) and DENV-4 (6.3% [4/64]). One IC (1.6%) was co-infected with both DENV-1 and DENV-2.

Out of the 130 ICs, 65 (50.0%) were hospitalized, of whom 62 recovered, 2 were palliatively discharged at the request of a family member, and 1 dropped out before the second sample collection (Table 1). The median hospitalization duration was 6 days (IQR: 5–8) (Table 1). The hospitalization rate was higher for ICs < 18 years of age (67.3% [35/52]) compared to those ≥18 years of age (38.5% [30/78]). For ICs < 18 years of age, the hospitalization rate was 71.4% (15/21) for those with primary infection and 64.5% (20/31) for those with post-primary infection. For ICs ≥ 18 years of age, the hospitalization rate was proportionally higher for ICs with primary infection (62.5%; 10/16) compared to those with post-primary infection (32.3%; 20/62).

**Figure 3:**
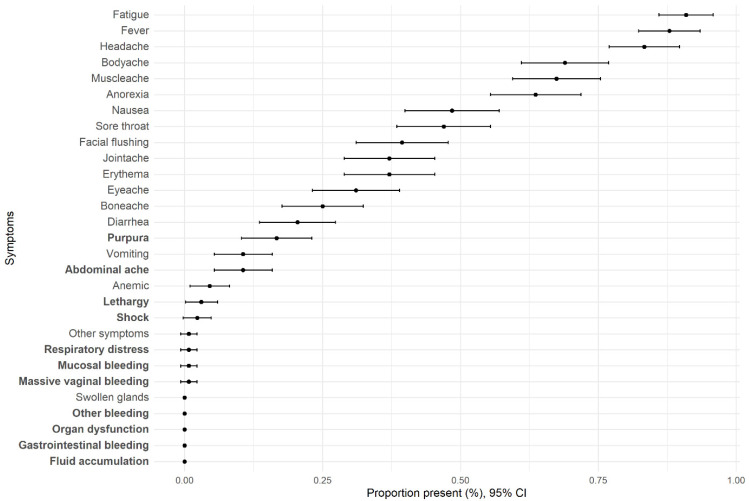
Baseline symptom proportions of index cases with 95% confidence intervals. Bold lettering indicates symptoms associated with dengue with warning signs (DWWS) and severe dengue (SD).

At enrollment, the most frequently observed symptoms in ICs (observed in ≥60%) were fatigue/malaise, fever, headache, body aches, muscle aches, and anorexia (Figure 3). Among the ICs, 29/130 (22.3%) exhibited dengue symptoms with warning signs, such as purpura, mucosal bleeding, abdominal pain, and lethargy. Additionally, 5/130 ICs (3.8%) displayed SD symptoms, including respiratory distress, shock, and/or massive vaginal bleeding (Table 1).

### 3.4. Characteristics and Dengue Infections in Household Contacts

The median age of HHCs was 35 years (IQR: 28–45), 42.9% (129/301) of HHCs were male, and the mean body mass index (BMI) was 22.4 (SD: 3.0). Among the enrolled HHCs, the detection of baseline anti-DENV IgG levels demonstrated a history of prior DENV infection for 87.0% (262/301), while 13.0% (39/301) were DENV-naïve at baseline. DENV-naïve individuals had a median age of 27 years (IQR: 21–31), while those with a history of DENV infection had a median age of 36 years (IQR: 30–48).

**Figure 4:**
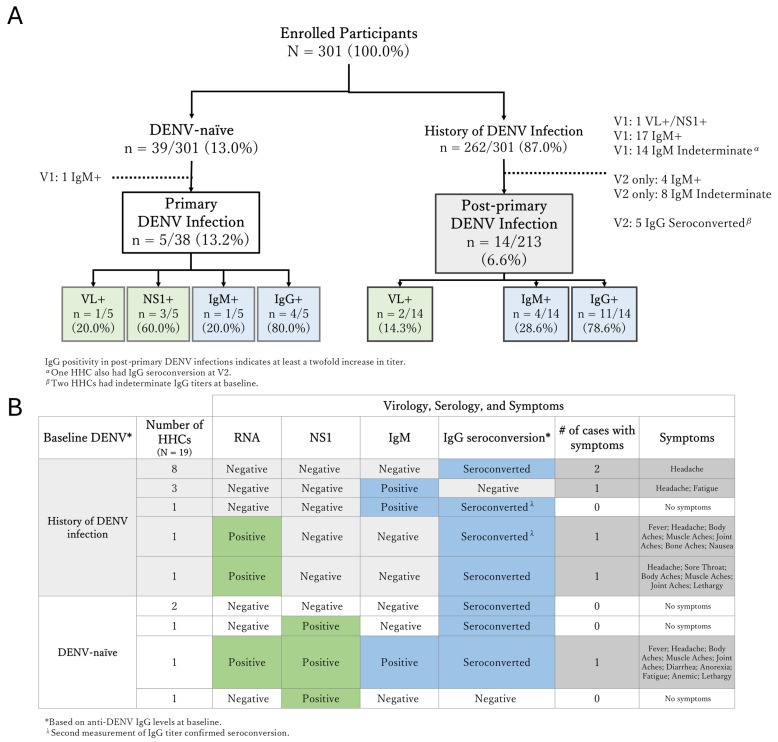
Flowchart of the immunological profiles and viral markers of household contacts (**A**), and table of DENV-positive cases (**B**). Virological positive cases are shown in green, and serological positive cases in blue.

Fifty HHCs (16.6%, 50/301) were excluded from the HHC infection analysis due to dengue infections at enrollment based on anti-dengue IgM positivity (22/301), anti-dengue IgM being indeterminate (22/301), anti-dengue IgG seroconversion at visit 2 (5/301), and DENV RNA+NS1 positivity (1/301). At the post-baseline follow-up visits, 2.0% of HHCs (5/251) had DENV infections based on virology parameters: two participants became positive for only DENV RNA, one participant for both DENV RNA and NS1, and two participants became positive for NS1 only. Anti-DENV IgG and/or IgM seroconversion was detected in 7.2% of HHCs (18/251) (Figure 4). Overall, during follow-up, 19/251 (7.6%) HHCs showed positivity or an increase in viral, serological, or both viral and serological markers of DENV infections. These included 5 HHCs who were previously DENV-naïve and thus experienced a primary DENV infection and 14 HHCs with baseline evidence of a history of DENV infection experiencing a post-primary infection.

Out of these 19 HHCs with positive DENV viral and/or serological markers during follow-up, 6 HHCs experienced symptoms—4 (1.6%, 4/256) had dengue without warning signs (DWoWS), 2 (0.8%, 2/256) had dengue with warning signs (DWWS), and none had SD—while 13 HHCs (5.1%, 13/256) remained asymptomatic. The asymptomatic-to-symptomatic ratio was 2.2:1.

A broad range of dengue virological (DENV RNA/NS1) and serological (anti-DENV IgM/IgG) patterns were observed among HHCs exposed to DENV during the study enrollment and follow-up period. A classic primary DENV infection was observed during follow-up in a DENV-naïve HHC, characterized by simultaneous increases in DENV RNA and NS1, followed by a rise in both IgM and IgG levels (Figure S2A). In the other HHCs with virological and/or serological evidence of DENV infections, more diverse profiles were observed: in three HHCs, only DENV RNA or NS1 was detected, followed by IgG seroconversion (n = 1 primary and n = 2 post-primary infections). In one HHC with a primary dengue infection, only DENV NS1 was detected, without any rise in serological markers, and in three HHCs with post-primary dengue infection, only IgM was detected, without virological markers or a rise in IgG. In one HHC, a rise in IgM and IgG seroconversion was observed. Furthermore, two HHCs with a primary infection and eight HHCs with a post-primary infection showed a prominent rise in IgG levels during the follow-up period, although no DENV RNA or NS1 could be detected (Figure S2B).

Among the 50 HHCs excluded from the infection analysis, 1 individual with detectable DENV RNA and NS1 at enrollment showed high initial levels of both markers, followed by a rise in IgG levels (Figure S2C).

## 4. Discussion

We conducted a prospective community-based study to assess dengue infections among HHCs of dengue ICs in a dengue-endemic setting in Vietnam. The majority of ICs (71.3%) completed RDT testing within 48 h of symptom onset. Early RDT testing correlated with NS1 positivity without anti-DENV IgM positivity in 75.4% of cases, indicating infection in the acute phase. In addition, our study demonstrated operational feasibility for the follow-up of HHCs to assess the incidence of DENV infections, with 92% (276/301) of HHCs successfully completing all 10 planned study visits, including sample collection at each visit. The intensive sample collection schedule, with twice-weekly collections, was designed to optimize the identification of dengue cases through the detection of DENV RNA and/or NS1, which can only be detected in the acute phase of the disease. DENV RNA or NS1 positivity, in general, may only be detected within 7 days after symptom onset. DENV viremia levels decrease rapidly following the onset of symptoms, and the window of DENV RNA positivity may vary with infecting serotype and/or immune status [16]. Our findings demonstrate the operational feasibility of identifying index cases within 72 h of symptom onset, enrolling their household contacts, and successfully completing intensive follow-up—insights that are significant for designing future community-based dengue intervention studies in endemic regions like Vietnam. These may include antiviral treatment studies, where rapid identification and intervention after symptom onset are essential, as well as preventive studies that require the early identification of at-risk individuals and intensive follow-up.

In this study, we reported the prevalence of dengue immunity among DENV-positive ICs and their HHCs in a dengue-endemic setting in Nha Trang, central Vietnam. Nha Trang city is a highly endemic dengue setting, with an incidence of clinically compatible dengue that was reported to be 2.8 per 1000 persons in a study conducted in 2011 [17]. We observed that prior dengue exposure increases with age, and by the age of 18 years, the majority of cases (80%) had been exposed to dengue at least once, classifying them as post-primary dengue cases. The primary dengue cases among adult ICs were younger than the post-primary dengue cases. However, the median age of ICs under 18 years did not differ notably between those with primary and post-primary dengue.

Among the enrolled HHCs, 13.0% were dengue-naïve, indicating that this population is at risk of a primary dengue infection in the community. As expected, the dengue-naïve HHCs were younger than the HHCs with a history of a prior DENV infection (median: 27 years vs. 36 years).

During follow-up of the HHCs, twice-weekly blood sampling revealed dengue infections in 2.0% based on viral markers (DENV RNA and/or NS1) and in 7.2% based on serological markers (anti-DENV IgM and/or IgG seroconversion). Notably, NS1 remained negative at the visits with testing for all HHCs with a history of prior DENV infection who experienced a post-primary DENV infection during the study. These results are consistent with findings from Kulkarni and colleagues, who reported lower NS1 titers in patients with secondary dengue infections compared to those with primary infections [18]. Among the 7.6% (19/251) of HHCs with viral and/or serological evidence of DENV infection, six were symptomatic, including four without warning signs and two with warning signs. Both symptomatic HHCs with warning signs were DENV RNA-positive. The remaining 13 HHCs with DENV infections were asymptomatic; none were DENV RNA-positive, but NS1 was detected in 2 of these cases. The incidence of asymptomatic dengue infections in HHCs in this study was 5.2% (13/251), with a ratio of asymptomatic-to-symptomatic cases of 2.2:1. This ratio was slightly higher than the 1.5:1 reported in a contact cluster study by Riswari and colleagues (2.2% incidence) [19].

Our results are consistent with those of a previous cross-sectional dengue serosurvey in Nha Trang [20], which found that approximately 7% of the study population tested IgM-positive for dengue, suggesting recent exposure to the virus. A community contact cluster design similar to our study was used in a prospective observational study conducted in Indonesia from 2005 to 2009. In that study, 3.7% of community contacts of dengue cases, followed up for 2 weeks, were diagnosed with acute DENV infections (DENV RNA-positive and/or IgM seroconversion) [21]. Unlike our study, the dengue ICs in this study were enrolled later in their acute infection, with a median fever duration of 5 days. The majority of the ICs in the study in Indonesia were negative for DENV RNA and IgM-positive at enrollment, while the majority of ICs enrolled in our study were NS1- and/or DENV RNA-positive at enrollment. We observed that 16.6% of HHCs showed evidence of asymptomatic DENV infections at the time of enrollment. Similarly, a study conducted among asymptomatic blood donors in Nepal in 2023 reported a 7.8% rate of asymptomatic recent dengue infection [21]. Therefore, following the results of our study, a design like the one used in our study could support both a treatment strategy with early detection of ICs, as well as post-exposure prophylaxis, among HHCs who are positive for DENV RNA, NS1, or IgM at enrollment, and pre-exposure prophylaxis, among HHCs with no evidence of DENV infection at enrollment.

In our study, DENV-2 and DENV-1 were the predominant serotypes, followed by DENV-4. We did not detect DENV-3 in our study. A recent study from southern Vietnam conducted in 2022–2023 also reported that DENV-2 was the most prevalent (71.9%), followed by DENV-1 (24.0%) and DENV-4 (4.2%). Similarly, DENV-3 was not detected in another study [22].

### Limitations

HHCs with positive anti-DENV IgM at baseline were, as per the protocol, excluded from the HHC infection analysis. In addition, the analysis did not include HHCs with indeterminate anti-DENV IgM at baseline or with IgG seroconversion at visit 2. This approach represents a more stringent analysis, also considering the potential cross-reactivity of the IgM ELISA with other flaviviruses such as Japanese encephalitis virus and zika virus, which were not tested for in this study. Although this may have led to missed post-baseline DENV infections among the HHCs who were excluded from the analysis, the overall outcome of the study was not affected. We did not perform anti-dengue neutralization antibody assays (PRNT) on the samples, limiting our ability to analyze serotype-specific immune responses. Dengue virus genomic data were not yet available, and different serotype variants may exhibit distinct clinical and immunological characteristics, which we were unable to investigate. Cytokine profiling and transcriptomic analysis were not included in this manuscript, but we plan to further investigate these in the near future.

## 5. Conclusions

This study demonstrated the operational feasibility of a dengue IC-HHC community-based study design, with the rapid identification of ICs, the enrollment of their HHCs, and successful completion of an intensive sampling follow-up of HHCs. With blood sampling twice a week, dengue infection was detected in 2.0% (based on virology) to 7.2% (based on serology), and 13/19 HHCs with dengue infection were asymptomatic. This may indicate that several parameters and/or more frequent sampling timepoints might be required to detect a dengue infection. The present study paves the way for future interventional studies evaluating anti-DENV agents for the treatment or prevention of dengue infections in the community.

## Figures and Tables

**Table 1:** Epidemiological and clinical characteristics of index cases.

	Index Cases < 18 years			Index Cases ≥ 18 years			
	Primary	Post-Primary	All < 18 Years	Primary	Post-Primary	All ≥ 18 Years	Total
**N**	21	31	52	16	62	78	130
**Age**							
Median (IQR)	10 (9–11)	10 (8–15)	10 (8–14)	26 (21–32)	33 (26–43)	31 (25–39)	23 (11–34)
**Sex, n (%)**							
Males	15 (71.4)	18 (58.1)	33 (63.5)	4 (25.0)	32 (51.6)	36 (46.2)	69 (53.1)
Females	6 (28.6)	13 (41.9)	19 (36.5)	12 (75.0)	30 (48.4)	42 (53.8)	61 (46.9)
**BMI**							
Mean (SD)	19.6 (3.8)	19.1 (4.2)	19.3 (4.0)	21.4 (2.2)	21.9 (3.1)	21.8 (3.0)	20.8 (3.6)
**Days from symptom onset to enrollment**							
Mean (SD)	1.8 (0.6)	2.0 (0.8)	1.9 (0.7)	1.8 (0.8)	2.4 (0.7)	2.3 (0.8)	2.1 (0.8)
**Disease severity ^α^, n (%)**							
Dengue without warning signs	13 (61.9)	23 (74.2)	36 (69.2)	8 (50.0)	52 (83.9)	60 (76.9)	96 (73.8)
Dengue with warning signs	8 (38.1)	6 (19.4)	14 (100.0)	7 (43.8)	8 (12.9)	15 (19.2)	29 (22.3)
Severe dengue	0 (0.0)	2 (6.5)	2 (100.0)	1 (6.3)	2 (3.3)	3 (3.8)	5 (3.8)
**Cases hospitalized, n (%)**	15 (71.4)	20 (64.5)	35 (67.3)	10 (62.5)	20 (32.3)	30 (38.5)	65 (50.0)
**Days of hospitalization**							
Median (IQR)	6 (4–8)	5 (4–6)	6 (4–7)	6 (5–7)	7 (6–10)	6 (5–9)	6 (5–8)
**Hospitalization outcome, n (%)**							
**N**	15	20	35	10	20	30	65
Hospitalized and recovered	15 (100.0)	20 (100.0)	35 (100.0)	10 (100.0)	17 (85.0)	27 (90.0)	62 (95.4)
Palliative discharge	0 (0.0)	0 (0.0)	0 (100.0)	0 (0.0)	2 (10.0)	2 (6.7)	2 (3.1)
Hospitalized and dropped out of the study	0 (0.0)	0 (0.0)	0 (100.0)	0 (0.0)	1 (5.0)	1 (3.3)	1 (1.5)

^α^ Dengue without warning signs: erythema, nausea, vomiting, headache, eye aches, facial flushing, body aches, bone aches, sore throat, fatigue, anemia, myalgia, and arthralgia. Dengue with warning signs: purpura, mucosal bleeding, abdominal pain, and lethargy. Severe dengue: respiratory distress, gastrointestinal bleeding, massive vaginal bleeding, and other bleeding.

## Data Availability

The original contributions presented in this study are included in the article/Supplementary Materials. Further inquiries can be directed to the corresponding author.

## Supplementary Materials

The following supporting information can be downloaded at https://www.mdpi.com/article/10.3390/v17060859/s1: Figure S1. Study enrollment and household contact follow-up flow (appendix); Figure S2. Classical primary dengue infection case (**A**), post-primary dengue exposure before enrollment (**B**), IgG seroconversion without DENV RNA/NS1 signals (**C**).
