# Supplementary material for: Dengue Infections Among Household Contacts of Symptomatic Index Cases: Implications for Community-Based Intervention Studies
Source: Viruses. 2025 Jun 17;17(6):859. doi: 10.3390/v17060859 (PMC12197545; doi:10.3390/v17060859)
Supplement: Supplementary file 1 [file viruses-17-00859-s001.zip › viruses-3689150-supplementary.pdf]

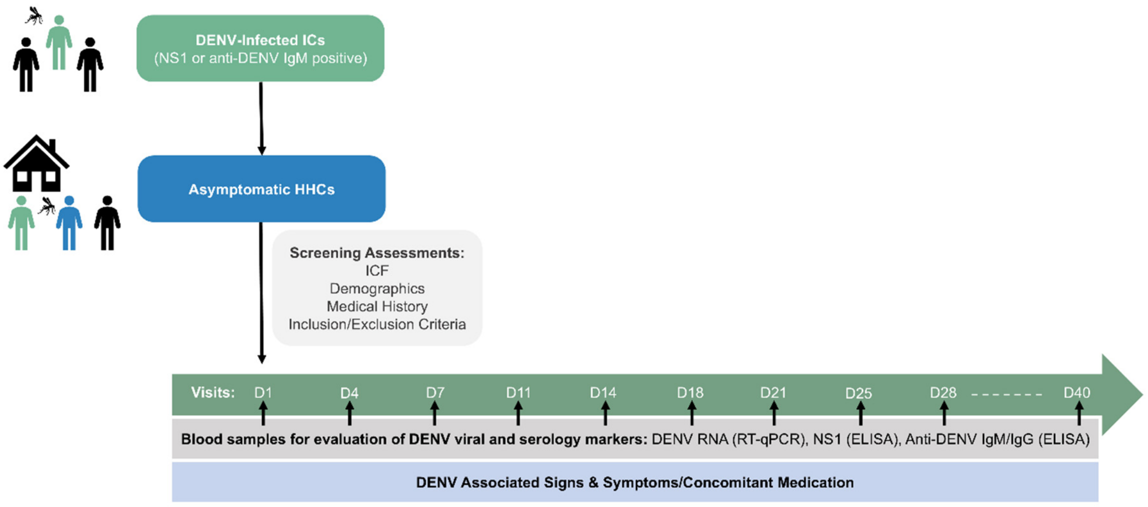

Figure S1. Study enrollment and household contacts follow-up flow (appendix)

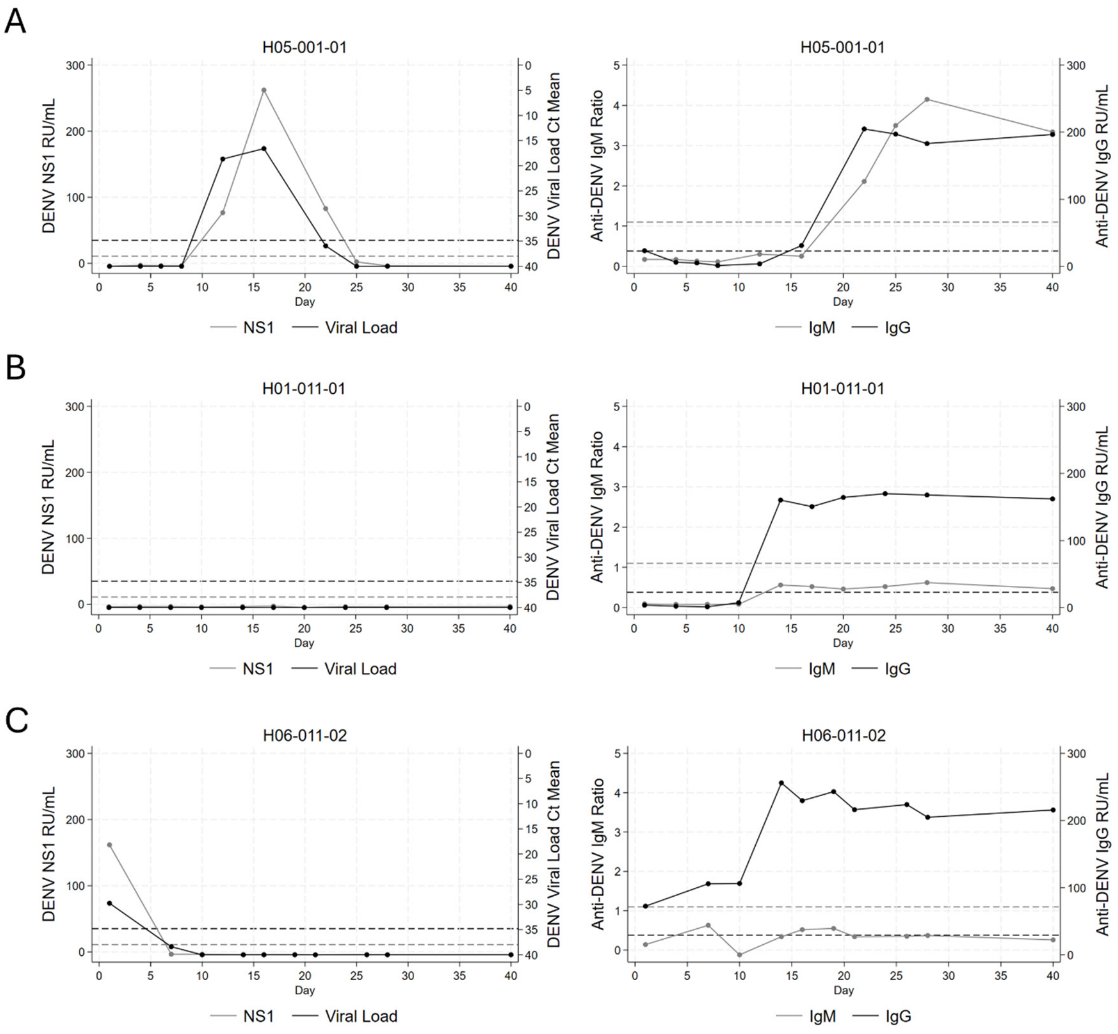

Figure S2. Classical primary dengue infection case (A), post-primary dengue exposure before enrollment (B), IgG seroconversion without DENV RNA/NS1 signals (C).
